# Carbon Dynamics, Development and Stress Responses in *Arabidopsis*: Involvement of the APL4 Subunit of ADP-Glucose Pyrophosphorylase (Starch Synthesis)

**DOI:** 10.1371/journal.pone.0026855

**Published:** 2011-11-03

**Authors:** Cécile Sulmon, Gwenola Gouesbet, Fanny Ramel, Francisco Cabello-Hurtado, Christophe Penno, Nicole Bechtold, Ivan Couée, Abdelhak El Amrani

**Affiliations:** 1 Centre National de la Recherche Scientifique, Université de Rennes 1, UMR 6553 ECOBIO, Rennes, France; 2 Institut National de la Recherche Agronomique, Unité de Génétique et d'Amélioration des Plantes, Centre de Versailles-Grignon, Versailles, France; Instituto de Biología Molecular y Celular de Plantas, Spain

## Abstract

An *Arabidopsis thaliana* T-DNA insertional mutant was identified and characterized for enhanced tolerance to the singlet-oxygen-generating herbicide atrazine in comparison to wild-type. This enhanced atrazine tolerance mutant was shown to be affected in the promoter structure and in the regulation of expression of the APL4 isoform of ADP-glucose pyrophosphorylase, a key enzyme of the starch biosynthesis pathway, thus resulting in decrease of *APL4* mRNA levels. The impact of this regulatory mutation was confirmed by the analysis of an independent T-DNA insertional mutant also affected in the promoter of the *APL4* gene. The resulting tissue-specific modifications of carbon partitioning in plantlets and the effects on plantlet growth and stress tolerance point out to specific and non-redundant roles of APL4 in root carbon dynamics, shoot-root relationships and sink regulations of photosynthesis. Given the effects of exogenous sugar treatments and of endogenous sugar levels on atrazine tolerance in wild-type *Arabidopsis* plantlets, atrazine tolerance of this *apl4* mutant is discussed in terms of perception of carbon status and of investment of sugar allocation in xenobiotic and oxidative stress responses.

## Introduction

Singlet oxygen is a major component of reactive oxygen species (ROS) dynamics in higher plants [1]. It has been shown to be involved in photo-induced damage of leaf tissues [2]. Moreover, various herbicides lead to singlet oxygen over-production, with resulting highly toxic effects [3]. For instance, atrazine (2-chloro-4-ethylamino-6-isopropylamine-1,3,5-triazine), which is a widely-used herbicide of the triazine class, inhibits photosystem II (PSII) by binding to the D1 protein, thus blocking electron transfer to the plastoquinone pool [3]. This prevents conversion of absorbed light energy into electrochemical energy and results in overproduction of triplet chlorophyll and singlet oxygen, oxidative stress and final bleaching [3]. Thus, herbicide treatments, as well as the use of specific mutants, such as the *flu* mutant of *Arabidopsis thaliana* [4], are useful to understand the complexity of ROS networks and the regulation of oxidative stress responses.

Atrazine tolerance can result from activation of detoxification mechanisms generally consisting in induction of catabolic pathways or conjugation to glutathione, both leading to stable and non-reactive compounds. Atrazine tolerance of populations of the maize weed *Setaria faberi* was thus shown to result from increase of monooxygenation reactions and glutathione-*S*-transferase activities [5]–[7]. Windsor et al. [8] also demonstrated that atrazine tolerance could originate from mechanisms of cell efflux by overexpression of a membrane transporter (*At*Pgp1) of the ATP binding cassette family in *Arabidopsis thaliana* transgenic lines. Finally, we have shown that sucrose and, to a lesser extent, glucose conferred atrazine tolerance to *Arabidopsis* plantlets [9]–[11]. Exogenous sugar treatment maintained PSII activity and phototrophic growth in the presence of atrazine concentrations that were otherwise lethal in the absence of exogenous sucrose. This induction of tolerance, which was also observed in *Arabidopsis* accessions exhibiting high endogenous sugar levels, could be ascribed to interacting effects of sucrose and atrazine on expression of stress-response genes, resulting in biochemical responses to stress and enhanced control of oxidative stress [12]–[14].

The T-DNA-mutagenized *Arabidopsis thaliana* collection (ecotype Wassilewskija,Ws) of the Institut National de la Recherche Agronomique (INRA, Versailles, France) [15] was screened in the presence of lethal concentrations of atrazine. Whereas atrazine treatments resulted in growth inhibition, oxidative injury and bleaching in wild-type (WT) plantlets, some T-DNA-mutagenized lines showed significant ability to grow and long-term survival under various conditions of atrazine exposure. The present work characterizes one such mutant, which presented, in addition to enhanced atrazine tolerance, enhanced root growth in the absence of atrazine. The T-DNA insertion is shown to be localised in the upstream region of the *APL4* (ADP-glucose pyrophosphorylase large subunit 4) gene (*At2g21590*), with consequences on *APL4* mRNA levels, carbon partitioning, shoot-root allocation and relationships, and carbohydrate accumulation. These effects are discussed in the context of sink and carbon/nitrogen regulation of photosynthesis and carbon fixation. Moreover, given the effects of exogenous sugar treatments [9], [12] and of endogenous sugar levels [14] on atrazine tolerance in wild-type *Arabidopsis* plantlets, atrazine tolerance of this *apl4* mutant is discussed in terms of perception of carbon status and of investment of sugar allocation in xenobiotic and oxidative stress responses, thus highlighting the specific and non-redundant roles of APL4, relatively to other ADP-glucose pyrophosphorylase (AGPase) subunits.

## Results

### Isolation and characterization of an *Arabidopsis* mutant showing enhanced tolerance to atrazine

The different lines of the T-DNA-mutagenized *Arabidopsis thaliana* collection (ecotype Wassilewskija (Ws)) of the Institut National de la Recherche Agronomique were grown on 1x Murashige and Skoog (MS)-agar in the presence of a lethal concentration of the singlet-oxygen-generating herbicide atrazine (500 nM). This concentration induces, in WT *Arabidopsis* plantlets, growth arrest and cotyledon bleaching within 10 days of growth [9]. Some mutant lines were found to maintain plantlet development in the presence of atrazine, thus showing a phenotype of *enhanced atrazine tolerance* (*eat*). Lines for which this phenotype was maintained in the T2 and T3 generations were kept for further analysis. One of these lines, named *eat1*, which showed significant atrazine tolerance, was further investigated.

**Figure 1:**
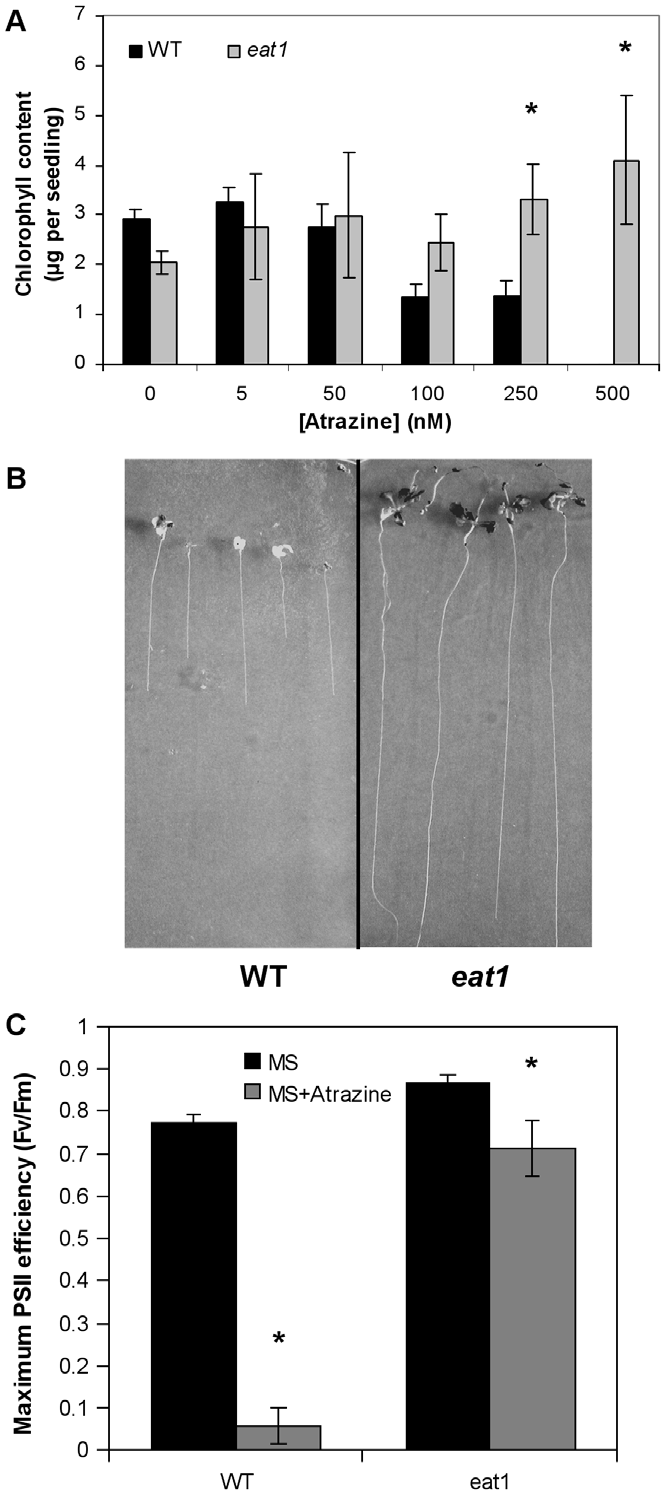
Characterization of atrazine tolerance in *eat1 Arabidopsis* mutant plantlets. Chlorophyll content (A), plantlet development (B) and PSII efficiency using Fv/Fm ratio (C) were compared between the *eat1* mutant (Ws genetic background) and the Ws ecotype (WT). Direct exposure to atrazine during germination and early growth (A) was carried out in the presence of varying concentrations of atrazine from 5 nM to 500 nM. Chlorophyll contents (A) were measured on 3 replicas of 2 to 5 pooled plantlets each and results are given as the mean (± S.E.M.) of these three determinations. In transfer experiments (B, C), germination and early development were carried out on 1x MS-agar medium, and 10-day-old plantlets were then transferred to 1x MS-agar medium containing 1 µM atrazine. Measurements were carried out after 15 days of further growth. Values of Fv/Fm (C) are the mean (± S.E.M.) of measurements on at least ten 25-day-old plantlets. Asterisks represent statistically significant differences (Mann-Whitney test, P<0.05) between WT and *eat1* (A) or between MS and MS+atrazine treatments (C). These experiments were carried out three times and results were similar.

In order to characterize the level of atrazine tolerance, *eat1* mutant plantlets were grown on 1x MS-agar medium in the presence of 5 nM to 500 nM atrazine. Whereas atrazine significantly decreased chlorophyll contents of WT plantlets at 100 nM, the chlorophyll contents of *eat1* plantlets were not affected by the herbicide at the concentrations tested (Figure 1A). Higher concentrations of atrazine, such as 1 µM, resulted in bleaching of *eat1* plantlets (data not shown), to the same extent as in the case of WT plantlets [9], when atrazine exposure started at the onset of germination. However, *eat1* plantlets, which had been initially grown in the absence of atrazine, were found to escape inhibition when transferred on medium containing 1 µM atrazine (Figure 1B, C), thus resulting in leaf development, photosynthesis activity, root growth and eventually floral bolt formation. This higher tolerance related to the mode of atrazine application was in line with previous studies [14]. In contrast, WT plantlets subjected to the same treatment showed inhibition of photosynthesis, growth arrest, and bleaching (Figure 1B, C).

**Figure 2 pone-0026855-g002:**
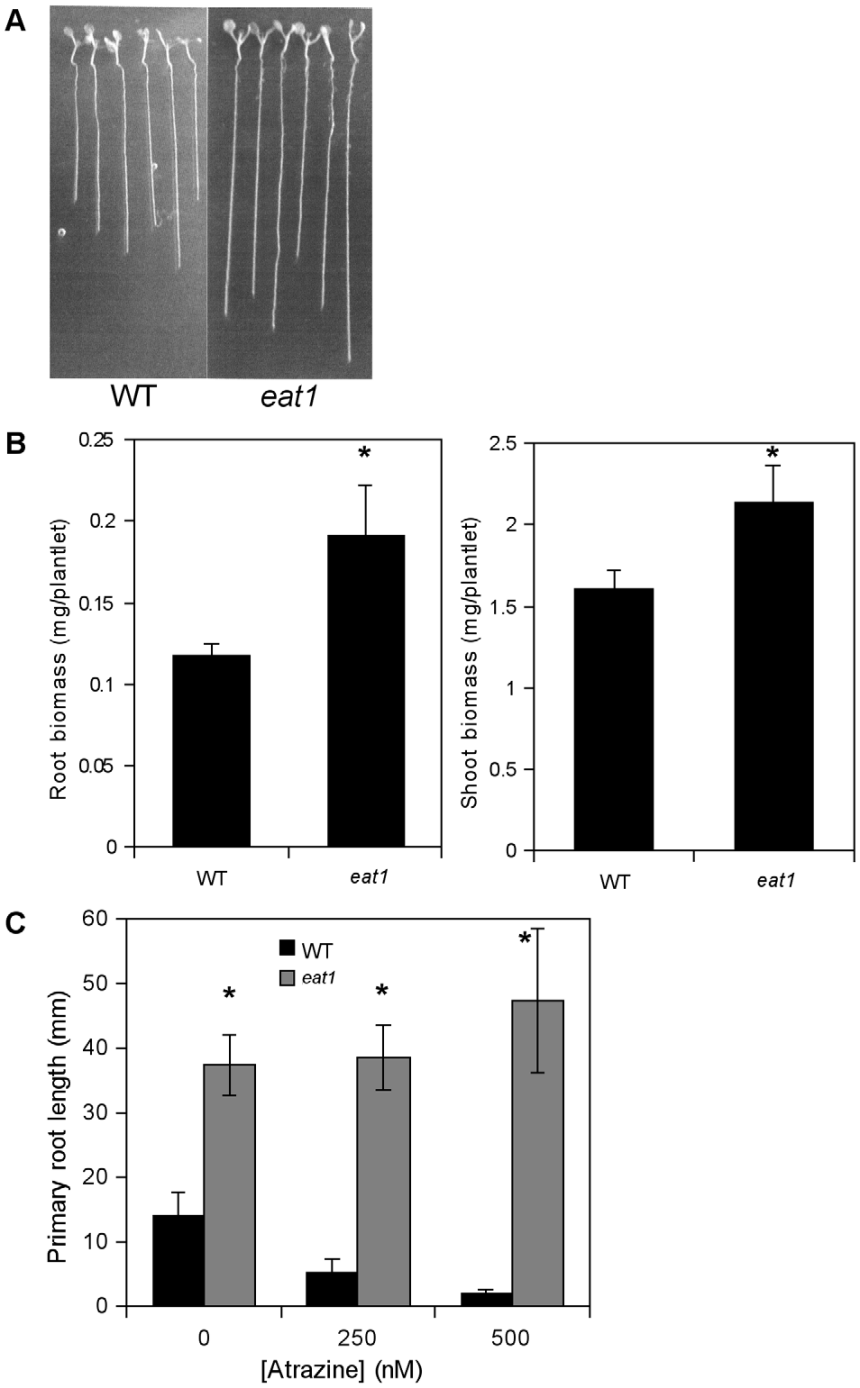
Plantlet growth of the *eat1 Arabidopsis* mutant. Plantlet development (A), shoot and root fresh weight (B) and length of primary roots (C) were compared between the *eat1* mutant (Ws genetic background) and the Ws ecotype (WT). Seeds of the *eat1 Arabidopsis* mutant line were germinated on 1x MS-agar medium in the absence (A, B) or in the presence of 250 nM to 500 nM atrazine (C). Plantlet development was carried out for 15 days. Values are the mean (± S.E.M.) of measurements on at least sixteen 15-day-old plantlets (B, C). Asterisks represent statistically significant differences (Mann-Whitney test, P<0.05) between WT and *eat1*. These experiments were carried out three times and results were similar.

The *eat1* mutant also exhibited a phenotype of enhanced root growth (Figure 2A), enhanced root biomass (Figure 2B), and enhanced leaf biomass (Figure 2B) in the absence of atrazine treatment. The root:shoot ratio (fresh weight/fresh weight) was found to increase from 0.0761 in Ws to 0.0894 in the *eat1* mutant. Finally, enhanced root growth of *eat1* was maintained under xenobiotic and oxidative stress conditions in the presence of sublethal (250 nM) and lethal (500 nM) herbicide concentrations (Figure 2C).

### Characterization of the T-DNA insertion in the enhanced-atrazine-tolerance mutant

**Figure 3 pone-0026855-g003:**
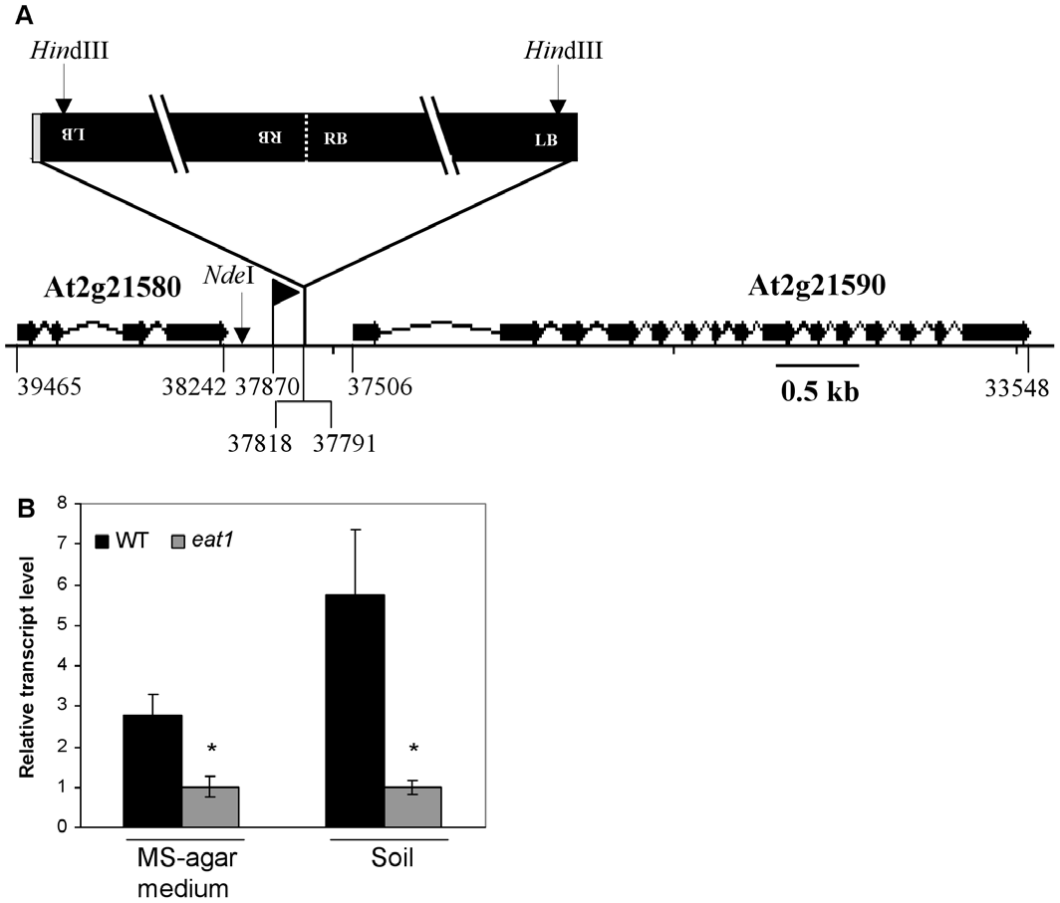
Characterization and effect on gene expression of the T-DNA insertion in the *eat1 Arabidopsis* mutant. T-DNA insertion was localised between the *At2g21580* and *At2g21590* genes (black bars), which encode, respectively, the 40S ribosomal protein S25, and an ADP-glucose pyrophosphorylase large subunit, recently characterized as APL4 [21] (A). The grey bar at the 5′ end of insertion represents a 21 bp unknown sequence and the flag corresponds to the BH755830 line [52]. The positions of the *At2g21580* and *At2g21590* genes, of the mutant line and of the T-DNA, on the F2G1 BAC clone are given. Expression of *At2g21590* gene in plantlets cultivated on MS-agar medium and on soil was analysed by real-time RT-PCR (B). Total RNA was isolated from 30-day-old plantlets grown on 1x MS-agar medium and from 5-week-old plantlets grown for 15 days on 1x MS-agar medium, transferred to soil and further grown under controlled conditions (16-h light at 22°C and 8-h dark at 18°C). The Ws ecotype was used as WT. *At2g21590* mRNA levels were normalized with respect to housekeeping genes *ubiquitin5* and *β–tubulin*. Values are the mean (± S.E.M.) of six measurements. Asterisks represent statistically significant differences (Mann-Whitney test, P<0.05) between WT and *eat1*. These experiments were carried out twice and results were similar.

Position of the T-DNA insertion in the genome of the *eat1* mutant was determined by isolating the genomic DNA fragments flanking T-DNA borders, using the PCR walking method described by Devic et al. [16]. Results of amplification product sequencing and of sequence alignments revealed that the T-DNA insertion was located on a genomic region of chromosome II, corresponding to bacterial artificial chromosome (BAC) clone F2G1, between the 37791 and 37818 positions, thus indicating that the T-DNA insertion had induced a 27 bp deletion in genomic DNA (Figure 3A). Moreover, sequencing results showed that an unknown sequence of 21 bp (GAATAGTTGTGTGCAAATATC) was inserted upstream of the T-DNA insertion at position 37818 of the F2G1 BAC clone. Genomic DNA sequences flanking both sides of the insertion corresponded to T-DNA left borders (LB), whereas only T-DNA sequence was amplified with primers corresponding to the T-DNA right border (RB), thus suggesting that the insertion consisted of two T-DNAs joined side-by-side in inverse orientation, so that only left borders were directed towards genomic DNA (Figure 3A). Southern blot analysis, using DNA probes corresponding to T-DNA LB and RB sequences, confirmed the localization and structure of the insertion previously suggested by sequencing results (Figure S1).

The T-DNA insertion was located downstream of a gene (*At2g21580*) encoding a 40S ribosomal protein S25 (RPS25B) and upstream of a gene (*At2g21590*) encoding a large subunit (APL4) of AGPase (Figure 3A). The *At2g21590* gene is one of the six AGPase subunit genes involved in the formation of AGPase heterotetrameric complexes that catalyse the synthesis of ADP-glucose from glucose-1-phosphate and ATP, and which constitutes the first step of starch biosynthesis in photosynthetic and non-photosynthetic organs [17]–[20].

### The enhanced-atrazine-tolerance mutant is affected in APL4 mRNA expression

The *At2g21580* gene was found to be similarly expressed in WT and in the *eat1* mutant (data not shown), thus indicating that the T-DNA insertion downstream of *At2g21580* (Figure 3A) did not affect expression of this gene. In contrast, *At2g21590* gene expression was significantly reduced in the *eat1* mutant in comparison to WT, whether in MS-agar or soil culture conditions (Figure 3B). These results demonstrated that the T-DNA insertion, located 285 bp upstream of the transcription start and 1165 bp upstream of the initiation codon (Munich Information Center for Protein Sequence), affected expression of the *APL4* gene.

**Figure 4:**
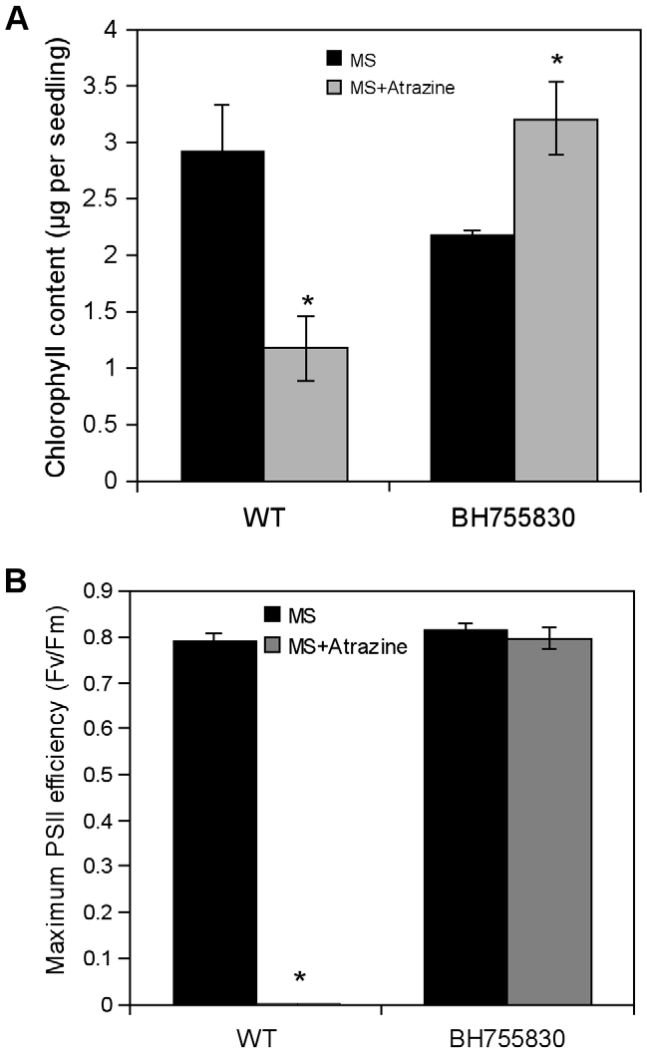
Phenotype characterization of the BH755830 T-DNA insertion line. The response to atrazine was measured using chlorophyll contents (A) and PSII efficiency (B) as markers. Germination and early development of WT (Col-0 ecotype) and BH755830 (Col-0 genetic background) were carried out on 1x MS-agar media, and 10-day-old plantlets were then transferred to 1x MS-agar media containing 1 µM atrazine. Measurements were done at the time of transfer (MS) and after 15 days of further growth (MS+Atrazine). Chlorophyll contents (A) were measured on 3 replicas of 2 to 5 pooled plantlets each and results are given as the mean (± S.E.M.) of these three determinations. Values of Fv/Fm ratio (B) are the mean (± S.E.M.) of measurements on at least twelve plantlets. Asterisks represent statistically significant differences (Mann-Whitney test, P<0.05) between chlorophyll content before transfer (MS) and at the end of transfer (MS+atrazine). These experiments were carried out twice and results were similar.

In parallel, a T-DNA-mutagenized line of the SIGnAL collection (Columbia ecotype, Col-0), BH755830, was found to present a T-DNA insertion at position 37870 in the F2G1 BAC clone, 52 bp upstream of the T-DNA insertion of the *eat1* mutant (Figure 3A). Phenotype characterization showed that 10-day-old plantlets of the BH755830 line exhibited, in comparison with the corresponding Col-0 WT background, the phenotype of atrazine tolerance when transferred on 1 µM herbicide, with maintenance of high chlorophyll level and photosynthetic activity (Figure 4). The BH755830 line also showed a phenotype of enhanced root biomass and shoot biomass (Figure S2). These results confirmed that observed phenotypes in the *eat1* mutant could be ascribed to the insertion in the upstream region of the *APL4* gene and to consequent modifications of gene expression (Figure 3B).

### Expression patterns of *APL3* and *APL4* genes in relation with source-sink relationships and with responses to abiotic stresses

AGPase is a heterotetrameric enzyme composed of two small (APS) and two large (APL) subunits. The *Arabidopsis* genome contains four *APL* genes (*APL1* to *APL4*) and two *APS* genes (*APS1*, *APS2*). While the main catalytic subunit is APS1 [18]–[21], the association of APL subunits with APS1 leads to the formation of functional AGPase complexes. In contrast, the APS2 subunit is considered to be inactive and unable to form functional AGPase complexes [20]. *APS2* may be a pseudogene [20]. Expression profiles of *APL* genes and regulatory properties of APL/APS heterotetramers have shown that APL subunits had specific tissue localizations and specific regulation roles [18]–[20]. APL1, and to a lesser extent APL2, are largely associated with source tissues and exhibit both regulatory and catalytic properties. In contrast, APL3 and APL4 subunits only exhibit a regulatory role and are mainly found in sink tissues, including roots.

Considering sink-related APL subunits, the APL4 (*At2g21590*) protein, which possesses a characteristic NTP-transferase domain (pfam 00483.11), is 84% identical to the sequence of APL3 (*At4g39210*), whereas it shows significantly lower homology to APL1 and APL2. However, in contrast with the protein sequence, the 5′ regulatory regions of *At2g21590* and *At4g39210* show significant divergence, with only 47% identity.

**Figure 5:**
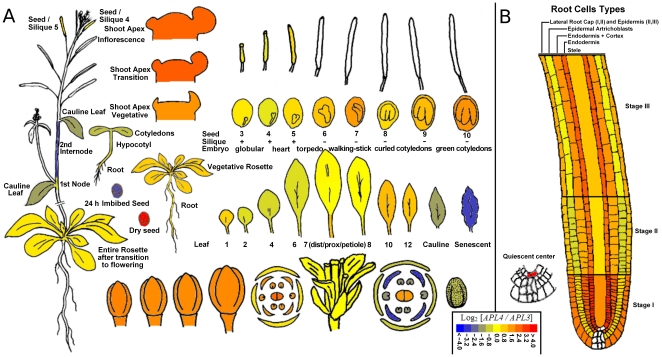
Electronic fluorescent pictography comparing relative expression levels of *APL4* and *APL3* genes. These pictures were obtained from the *Arabidopsis* eFP Browser tool at the Bio-Array Resource for Plant Biology website, using Developmental Map (A) and Tissue-specific (B) data series as data sources, and a threshold of 4.0. Colour scale represents Log_2_(transformed ratio of *APL4* relative to *APL3* signals).

Electronic fluorescent pictography, comparing relative expression level of *APL4* gene to that of *APL3* gene, was carried out using *Arabidopsis* Developmental Map [22] and Tissue-specific [23], [24] microarray data series (Figure 5). *APL3* and *APL4* genes exhibited distinct temporal and tissue-specific expression patterns in *Arabidopsis* plants. Moreover, *APL4* gene expression seemed to be preferentially associated with root and shoot apex sink tissues in comparison to *APL3* gene (Figure 5).

Expression of *APL3* and *APL4* genes under conditions of light treatments, of abiotic stress and of chemical stress was also investigated using Pearson correlation coefficients (Table 1). Considering light treatment, photoperiod regime did not seem to influence strongly the regulation pattern of *APL3* and *APL4* genes (Table 1). For the three conditions tested, long day (16 h light), short day (8 h light) and 12 h light, coregulation of expression was observed with a Pearson correlation coefficient varying between 0.67 and 0.86. In contrast, abiotic and chemical stress treatments resulted in various regulation patterns. In shoot, *APL3* and *APL4* gene expression exhibited overall positive correlation (Table 1). Except for oxidative stress and cold stress (correlation coefficient close to 0), the two genes were weakly (wounding, drought, and UV-B stress) to highly coregulated, with correlation coefficients (0.95–0.97), that reflect similar expression patterns under salt stress and under osmotic stress (Table 1). Regulation of expression of *APL3* and *APL4* genes was different in root tissues. Whereas these genes appeared to be partially coregulated under conditions of osmotic, oxidative and heat stress, they mainly exhibited inverse or independent regulation patterns. In particular, complete inverse regulation of *APL3* and *APL4* genes was found under conditions of genotoxic stress, not only in roots, but also in shoots (Table 1). Finally, photosynthesis inhibitor treatment on whole seedlings gave a correlation coefficient of −0.86 [59], thus also reflecting inverse regulation of *APL3* and *APL4*. These results therefore indicated that *APL3* and *APL4* genes may play non-redundant roles in source-sink relationships and in responses to abiotic stress.

**Table 1:** Pearson correlation coefficients of *APL4* to *APL3* gene expression vectors in response to light treatments and to abiotic and chemical stresses.

Photoperiod	16 h	8 h	12 h						
Seedlings	0.67	0.86	0.76						

Stress	Salt	Osmotic	Oxidative	Heat	UV-B	Drought	Cold	Wounding	Genotoxic
Root Shoot	−0.04	0.23	0.40	0.40	−0.16	−0.32	−0.41	−0.41	
	0.97	0.95	−0.03	0.68	0.37	0.31	−0.05	0.18	

Gene expression data were extracted from the publicly available database of the AtGenExpress Consortium. Pearson correlation coefficients of *APL4* to *APL3* gene expression were calculated using the Light series from Bläsing et al. [60] and Michael et al. [61], and the Abiotic Stress [59] and Chemical [59] series from the AtGenExpress Consortium data. Vector values are log_2_(transformed ratio of gene expression level relative to its control value) for each photoperiod or stress experiment.

Analysis of *APL3* and *APL4* gene expression in the context of atrazine sensitivity and tolerance was also studied in the Col-0 ecotype (Table 2). Whereas the *APL3* gene exhibited a significant plasticity of expression in response to sucrose and atrazine 24 h-treatments, *APL4* transcript levels did not show any differential expression. In fact, *APL3* transcript level increased in response to sucrose supply, and inversely decreased in the presence of atrazine (Table 2), which could correspond to redirection of carbon fluxes from starch synthesis to soluble sugars in relation with the development of stress responses [12]–[14]. However, the plasticity of *APL3* expression did not seem to compensate the decrease of *APL4* mRNA levels insofar as the *apl4* regulatory mutants (*eat1*; BH755830) showed significant phenotypic traits (Figure 1–4; Figure S2), thus confirming that *APL3* and *APL4* played distinct, non-redundant roles.

**Table 2:** Microarray analysis of *APL3* and *APL4* gene expression in response to atrazine and sucrose treatments.

Accession number	Gene product	Expression comparison log_2_(expression ratio)		
		Suc/Mtl	Mtl-Atrazine/Mtl	Suc-Atrazine/Mtl
*At4g39210*	APL3 : Large non-catalytic regulatory subunit	1.23	−1.15	−1.19
*At2g21590*	APL4 : Large non-catalytic regulatory subunit	not differentially expressed	not differentially expressed	not differentially expressed

Microarray data for *APL3* and *APL4* were extracted from our previous work [12], which was carried out under similar conditions of plant growth and plant treatment as those described in Materials and Methods. The characterization of *At4g39210* and *At2g21590* gene products was derived from previous studies [20], [21]. *Arabidopsis* plantlets (Col-0 wild ecotype) were transferred to MS-agar medium in the presence of 80 mM mannitol, 80 mM sucrose, 80 mM mannitol and 10 µM atrazine, or 80 mM sucrose and 10 µM atrazine. Transcriptome analysis was carried out on pairwise comparisons [12]. Relative expressions of genes after 24 h of treatment are given as their log_2_(expression ratio) for sucrose versus mannitol (Suc/Mtl), mannitol plus atrazine versus mannitol (Mtl-Atrazine/Mtl) and sucrose plus atrazine versus mannitol (Suc-Atrazine/Mtl) comparisons. Statistical analysis was carried out as previously reported [12]. Genes with a Bonferroni P-value higher than 5% were considered as being not differentially expressed [63].

### Effects of the *apl4* regulatory mutation on carbohydrate allocation and accumulation

**Figure 6:**
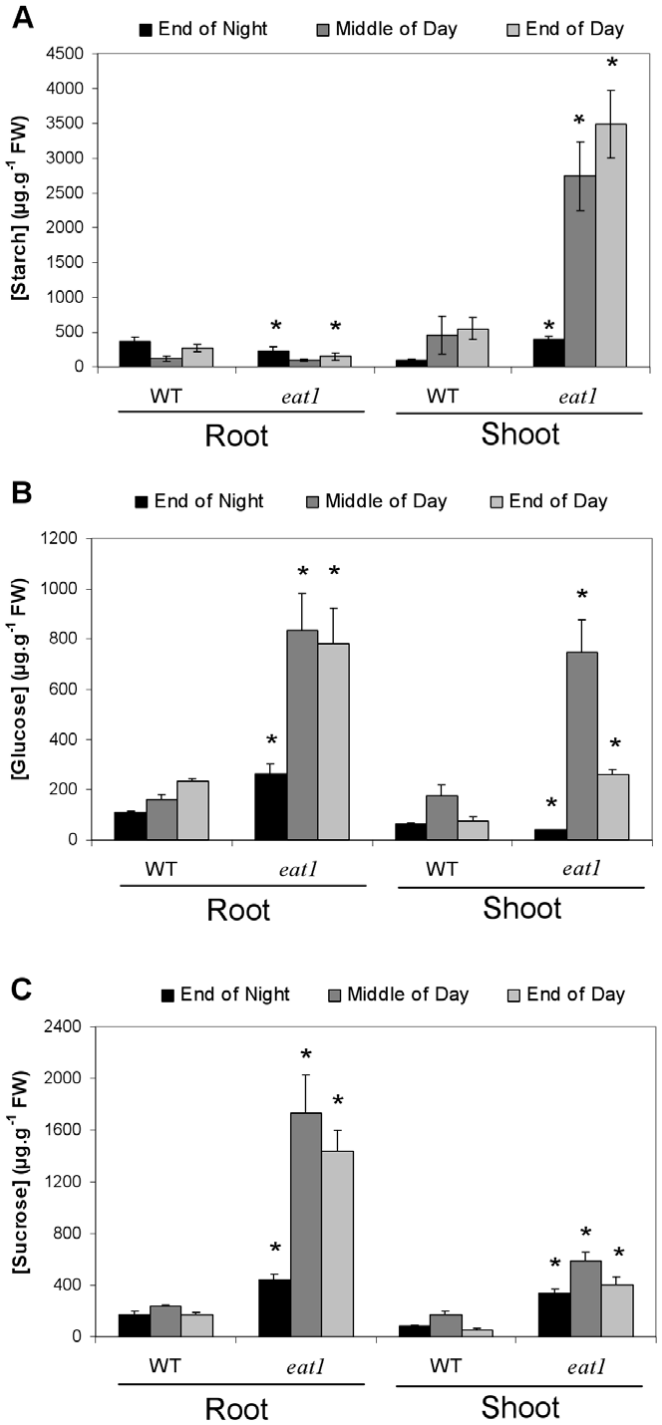
Carbohydrate partitioning in the *eat1 Arabidopsis* mutant. Starch (A), glucose (B), and sucrose (C) contents (µg.g^−1^ fresh weight (FW)) were quantified in roots and shoots, at the end of the night (End of Night), at the middle of the day (Middle of Day), and at the end of the day (End of Day). Seeds of WT (Ws ecotype) and *eat1 Arabidopsis* lines were germinated on 1x MS-agar medium and plantlet development was carried out for 15 days under a 12-h light period regime. Results are the mean (± S.E.M.) of three measurements realized on three independent extracts of root and shoot samples from at least 20 WT or *eat1* plantlets each. Asterisks represent statistically significant differences (Mann-Whitney test, P<0.05) between WT and *eat1* at a given time of measurement.

Carbohydrate partition and organ allocation were compared in 15-day-old plantlets of the *eat1* mutant and of the corresponding WT (Ws background). Plantlets were grown under a 12-h light period regime in order to increase the difference of starch-related phenotype between WT and mutant lines [25], [26]. Such light conditions did not influence the regulation of expression between *APL4* and *APL3* genes (Table 1). Starch, glucose and sucrose levels were determined at three time points of the photoperiod, i.e. at the end of the night (End of Night), in the middle of the day (Middle of Day), and at the end of the day (End of Day), separately in roots and in shoots (Figure 6).

Considering roots, the *apl4* regulatory mutant (*eat1*) showed a significant decrease of starch accumulation (Figure 6A), in accordance with the expression of *APL4* gene in roots (Figure 5), with the involvement of APL4 subunit in the AGPase complexes of sink tissues [18]–[20] and with the low-starch phenotypes shown by other *apl* mutants, such as *adg2-1*, which is affected in the *APL1* gene [27], [28]. Although the photoperiod-related pattern of starch dynamics remained similar to that of WT, starch levels in roots of the *eat1* mutant were significantly weaker at the end of the night and at the end of the day, thus confirming the involvement of APL4 in starch biosynthesis within root tissues (Figure 6A). These low levels of starch were concomitant with a significant increase of glucose and sucrose levels in roots of the *apl4* regulatory mutant (*eat1*), in comparison with those of WT plantlets, whatever the time of the photoperiod. This increase was stronger during the light period than at the end of night, in parallel with the extent of starch decrease (Figure 6).

In contrast, the shoots of the *eat1* mutant showed much higher levels not only of sucrose and glucose, but also of starch, in comparison with shoots of WT plantlets, especially during the light period (Figure 6). The photoperiod-related dynamics of these compounds remained similar to that of WT.

Thus, major modifications resulting from the *apl4* regulatory mutation, in comparison with WT plantlets, consisted in changes of starch-sucrose partition between roots and shoots in plantlets. However, quantitatively, decrease of starch levels in roots was associated with a 6-fold increase of starch levels in shoots during the light period, concomitantly with a large increase of sucrose and glucose levels in roots and in shoots. In particular, *eat1* mutant roots exhibited an 8-fold increase of sucrose levels in the light period (Figure 6). Considering the overall levels of carbohydrates in both roots and shoots, the *eat1* mutant thus showed a phenotype of enhanced carbohydrate accumulation in comparison with WT (Figure 6).

### Relationships between modifications of starch synthesis and atrazine stress responses

**Figure 7:**
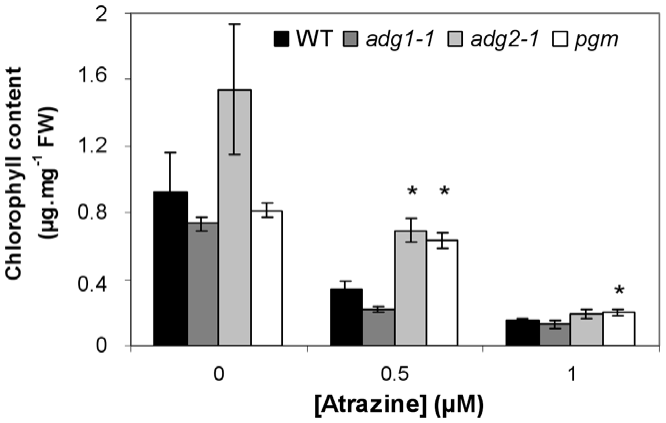
Effects of atrazine on chlorophyll levels of *adg1-1*, *adg2-1* and *pgm* mutants. The response to atrazine was measured using chlorophyll content (µg.mg^−1^ fresh weight (FW)) as marker. Seeds of the WT (Col-0 ecotype) and of the *adg1-1*, *adg2-1* and *pgm* starch-deficient or low-starch mutants (Col-0 genetic background) were germinated on 0.5x MS-agar medium [34] in the absence or presence of varying concentrations of atrazine from 0.5 µM to 1 µM. Plantlet development was carried out for 15 days under a 12-h light period regime. Chlorophyll contents were measured on 4 replicas of 2 to 5 pooled plantlets each and results are given as the mean (± S.E.M.) of these four determinations. Asterisks represent statistically significant differences (Mann-Whitney test, P<0.05) relatively to WT at a given atrazine concentration. These experiments were carried out twice and results were similar.

Both the *eat1* mutant and the BH755830 line showed enhanced tolerance to atrazine exposure (Figure 1 and Figure 4), under conditions where atrazine treatment causes a major oxidative stress resulting from singlet oxygen production [13]. In accordance with the effects of exogenous sucrose [9], [12] and of natural variation of endogenous sugars [14], the phenotype of enhanced atrazine tolerance could have been due to modification of carbon dynamics resulting in higher levels of soluble sugars. Other starch synthesis mutants, such as *adg1-1* [29], [30], *adg2-1* [27], [28] and *pgm* [31], which are respectively mutated in the *APS1* gene, the *APL1* gene and the *PHOSHOGLUCOMUTASE* gene, were subjected to atrazine stress (Figure 7). Chlorophyll levels were used as marker to monitor the effects of atrazine stress, as described previously [9], [13]–[14]. The behaviour of the *adg2-1* and *pgm* mutants confirmed that decrease of starch synthesis and increase of soluble sugars [25], [27], [31] were related to enhanced atrazine tolerance. However, this was not the case for the *adg1-1* mutant (Figure 7).

## Discussion

The enhanced atrazine tolerance phenotype of the *eat1* mutant and of the BH755830 line could be ascribed to insertional mutations in the promoter region of the *At2g21590* gene (Figure 3), which encodes the APL4 large subunit of AGPase [18]–[20].

**Figure 8:**
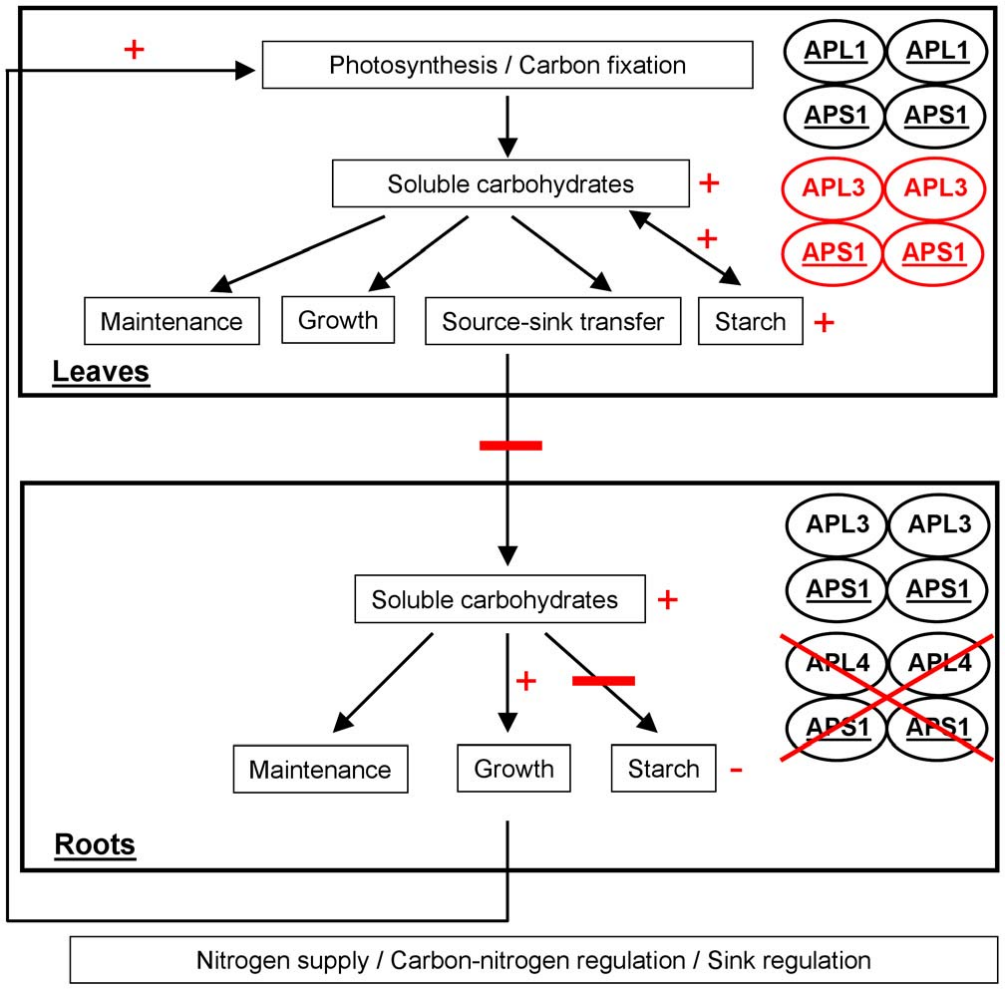
Hypothetic scheme of carbon allocation dynamics and source-sink relationships in the *eat1* mutant. This scheme is based on the characterisation of AGPase subunits by Crevillén et al. [19] and Ventriglia et al. [20]. The major heterotetrameric structures of ADP-glucose pyrophosphorylase in leaves and in roots are shown. The catalytic subunits [20] are underlined. Direct and indirect consequences of the *apl4* regulatory mutation are highlighted as red bars and minus signs indicating inhibition or decrease and as plus signs indicating activation or increase. The modifications affecting AGPase complexes in the *eat1* mutant are in red. The potential sink-source feedback regulation between roots and leaves is hypothesised from Paul and Foyer [45] and Martin et al. [46].

In higher plants, AGPase complexes are heterotetrameric combinations of small and large subunits presenting catalytic or regulatory functions and tissue specialisation [18]–[20]. The small APS subunits are clearly not redundant, with APS1 being catalytic in all plant tissues, and APS2 appearing to be non-functional [18], [19]. It has been recently shown that APL1 and APL2 subunits were also clearly distinct from APL3 and APL4 subunits on the basis of catalytic and regulatory properties and of differential involvement in starch synthesis between source (leaf) and sink (root) tissues [20], [32]. Moreover, the four genes encoding large subunits, *APL1* to *APL4*, clearly exhibit different expression patterns depending on organ, cell type and culture conditions [19], [33], [34]. Crevillén et al. [19] have thus shown that *APL1* was the most highly expressed *APL* gene in leaves, whereas *APL3* and *APL4* genes were the most highly expressed *APL* genes in roots. Nevertheless, some functional compensations are possible since analysis of *adg2-1* mutant showed that trehalose induction of *APL3* could complement *APL1* deficiency in leaves [34]. The present characterization of the *eat1* mutant and the comparison of microarray data (Figure 5, Tables 1 and 2) show that *APL3* and *APL4* are not redundant, in terms of expression patterns, despite the important plasticity of *APL3* expression (Table 2) [34]. The consequences of the *eat1* mutation on the functioning of AGPase subunits are summarized in the hypothetic scheme of Figure 8. This scheme is based on the present characterization of the *eat1* mutant and on existing literature [19]–[20].

AGPase is a key step in the regulation of photoassimilate partitioning [35] and consequently of carbon allocation within the plant [17]–[20], [36]. Thus, mutations of key components of AGPase [27]–[30], or of chloroplastic phosphoglucomutase [31], result in significant modifications of assimilate partitioning and carbon allocation, with starch deficiency and increased levels of soluble sugars [25], [31]. A similar trend of starch deficiency and high-sucrose content, and to a lesser extent high-glucose content, was found in the roots, but not in leaves, of the *eat1* mutant (Figure 6). These results thus associated *APL4* gene with the functioning of a sink tissue, in line with previous studies [18]–[20], and highlighted its important and non-redundant role in roots. Moreover, they suggested that, in accordance with expression patterns (Figure 5), *APL3* and *APL4* genes and/or subunits may not be submitted to similar regulation, and that the APL4 subunit significantly contributed to the regulation of root starch biosynthesis. Such differential involvement of *APL3* and *APL4* genes, confirmed by data of Tables 1 and 2, was consistent with significant divergence in their 5′ regulatory regions (data not shown). However, further work will be required to determine whether *APL4* is associated with specific cell types, and to what extent specific regulations controlling its expression are important relatively to common *APL3*/*APL4* regulations [19]. Finally, these changes of carbon partitioning and the consequent increase of soluble carbohydrates could be related to the increase of root growth in the *eat1* mutant (Figure 2). Decrease of storage function and enhanced carbon allocation to the roots has indeed been hypothesised to drive root growth, as shown in the case of the *adg1-1*, *adg2-1* and *pgm* starch-deficient mutants and by the effects of trehalose on AGPase activity and plantlet growth [33], [34], [37].

In parallel with the modifications of carbon partitioning in roots, the *eat1* mutant showed strikingly high levels of photoassimilates (starch, sucrose, glucose) in leaves (Figure 6). On the one hand, the absence of starch decrease in shoots of *eat1* mutant and the contrast with the situation in roots was consistent with the involvement of APL1 and APL2 large subunits in source tissues [18]–[20], which should not be affected by the mutation of the *APL4* gene. On the other hand, the increase of carbon assimilates (starch, sucrose, and glucose) in shoots indicated strong interactions between root and shoot carbon dynamics and an indirect impact of the *apl4* regulatory mutation on carbohydrate accumulation and carbohydrate allocation at whole plantlet level.

Chiou and Bush [38] have shown that sucrose treatment through the xylem sap acted as a signalling molecule leading to strong decrease of phloem loading and of carbohydrate allocation to the roots. The discrepancy between roots and shoots in the *apl4* regulatory mutant (*eat1*) may thus be a consequence of increased soluble carbohydrates in roots leading to decreased allocation from shoots to roots and overaccumulation of photoassimilates in leaves in the presence of active APL1/APS1 AGPase complexes (Figure 8). Moreover, Crevillén et al. [19] showed that long-term treatment with 100 mM exogenous sucrose highly induced *APL3* and *APL4* expression in leaves. The increase of starch accumulation in leaves of the *apl4* regulatory mutant (*eat1*) may therefore be due to the induction of the *APL3* gene in response to higher endogenous levels of glucose and sucrose and formation of APL3/APS1 complexes besides APL1/APS1 complexes, as summarized in the hypothetic scheme of Figure 8. This would also suggest that *APL3* and *APL4* play redundant roles in source tissues, in contrast with the situation of non-redundancy in roots. Taken together, these results therefore indicate that *APL4* is particularly important for regulating starch/sucrose partitioning in roots and that both *APL3* and *APL4* play important roles in carbon allocation.

Besides transcriptional regulations, changes of starch-sucrose-glucose partition in *eat1* mutant may also cause sugar-induced post-translational modifications of AGPase complexes [39]. In particular, high levels of sucrose or glucose have been found to induce redox activation of AGPase through, respectively, SnRK1-and HXK-dependent signalling pathways [40]–[42]. Reversible phosphorylation of APS and APL subunits has been hypothesized to regulate AGPase activity [39]. The SNF1-related kinase SnRK1, as protein-kinase and as key component of sugar signalling pathways, could be a common element for all these steps of starch synthesis regulation. The high-sucrose and low-starch partition in roots of *eat1* mutant may also reflect the role of APL4 in such post-translational regulations. This potential role will require further analysis.

The phenotype of highly-enhanced carbohydrate accumulation in both roots and shoots of the *eat1* mutant (Figure 6), associated with enhanced root and shoot biomass (Figure 2), may indicate enhanced photosynthesis and carbon fixation. *Arabidopsis* transgenic lines expressing antisense chloroplastic fructose-1,6-biphosphatase show similar pattern of carbohydrate accumulation, with plantlets exhibiting high levels of starch, sucrose and total sugars, and concomitant increased level of photosynthetic rate [43]. However, high levels of soluble sugars in leaves are expected to regulate negatively carbon assimilation-related processes [44]. On the other hand, increased root growth of the *eat1* mutant (Figure 2) may reflect modifications of the perception of carbon/nitrogen balance at root level, and, under conditions of high nitrogen availability given by the MS medium, increased root growth is in turn likely to improve nitrogen nutrition, with changes of shoot-root signalling resulting in enhancement of photosynthesis and carbon fixation [45], [46]. Thus, nitrogen supply in roots increases the levels of cytokinins, which move in the transpiration stream from roots to shoots and stimulate photosynthesis gene expression [45]. The positive effects of *apl4* mutation at root level on carbohydrate accumulation at shoot level (Figure 6) could be ascribed to this kind of root-shoot interaction, which has been indicated in the hypothetic scheme of Figure 8. It was also likely that the integration of all of these effects affecting metabolism, growth and development was related to the increase of root:shoot ratio in the *eat1* mutant (Figure 2B). APL4 activity and starch partitioning in roots may therefore play important roles in the connections between carbon/nitrogen perception at root level, root growth and root regulation of photosynthesis [45].

Variations of carbon supply and allocation have been shown to be involved in abiotic stress responses [47], especially through carbohydrate-modulated induction of defence-related genes [12], [48]–[50]. In the present work, enhanced tolerance to atrazine-mediated stress was shown not only by structural mutants of starch synthesis (*adg2-1*, *pgm*), but also by both *apl4* regulatory mutants, *eat1* and BH755830. This enhanced tolerance to atrazine-mediated stress could be ascribed to increased levels of soluble carbohydrates in these mutants [25]–[27], [29], [31] (Figure 6), in accordance with the effects of exogenous sucrose on atrazine stress responses [9], [12]–[13] and with the enhanced atrazine tolerance of high-sucrose natural accessions [14]. However, the reason why the *adg1-1* starchless mutant did not show any enhanced tolerance to atrazine in contrast with the *pgm* mutant remains unknown. Further work will be required to determine whether these different mutants of starch synthesis and starch-sucrose partitioning differ in the spatio-temporal dynamics of soluble carbohydrates and how they differ in terms of transcriptome profiling. The involvement of APS1, which is encoded by ADG1 [30] in other functions related to regulation of antioxidant responses could also be envisaged. This should contribute to understand which sugar and stress signalling pathways are perturbed in the various starch-synthesis mutants and explain the differential effects on stress tolerance. The relationships between carbon partitioning, carbon/nitrogen balance, shoot-root development and stress responses are likely to be important for plant breeding in the context of global change and of rising demands for food, bioenergy and ecological engineering [14], [51]. The characteristics of the *eat1* mutant indicate that novel regulations of root:shoot ratios remain to be investigated and that for instance the regulatory subunits of AGPase and the mutations and polymorphisms affecting their regulation could be interesting targets of plant breeding.

## Materials and Methods

### Plant material and growth conditions

Seeds were surface-sterilized for 5–10 min in 50% bayrochlore/50% ethanol, rinsed twice in absolute ethanol and dried overnight. Surface-sterilized seeds were plated on square Petri dishes for germination, and growth was carried out under axenic conditions. Petri dishes were sealed with Parafilm and placed in a cold chamber at 4°C during 48 h in order to break dormancy and homogenize germination. Petri dishes were then transferred to a control growth chamber and placed vertically at 22°C under a 16-h light period regime at 85 µmol m^−2^ s^−1^ unless otherwise specified. Seed germination and plantlet growth took place directly in the media under study, unless otherwise specified. Growth medium consisted of 0.8% (w/v) agar in 0.5x or 1x Murashige and Skoog (MS) basal salt mix (M5519, Sigma, www.sigmaaldrich.com) adjusted to pH 5.7. After dissolution in appropriate diluted-MS basal salt mix, atrazine was sterilised by microfiltration through 0.2 µm cellulose acetate filters (VWR, http://fr.vwr.com) and axenically added to melted agar-MS medium prior to pouring into Petri dishes. Atrazine stress experiments consisted in direct exposure to atrazine during germination and early growth or in transfer experiments where 2-week-old plantlets were transferred to atrazine-containing medium, as described in Ramel et al. [14].

The *apl4* regulatory mutant was obtained by screening of the T-DNA-mutagenized *Arabidopsis thaliana* collection [ecotype Wassilewskija (Ws)] of the Institut National de la Recherche Agronomique (INRA, Versailles, France) [15] on 1x MS-agar in the presence of lethal concentrations of atrazine. Mutant plantlets were then transferred to soil and grown under controlled conditions (16-h light at 22°C and 8-h dark at 18°C) to ensure seed set. The EMBL accession BH755830 line is an *Arabidopsis* T-DNA-mutagenized line of the Columbia (Col-0) ecotype [52] and was obtained from the Nottingham *Arabidopsis* Stock Centre (NASC, http://arabidopsis.info; NASC ID: N552250; SALK_052250). The *pgm* mutant was kindly provided by Dr Yves Gibon (INRA Bordeaux, University of Bordeaux 1&2, UMR 619 Fruit Biology, Villenave d'Ornon, France). The *adg1-1* and *adg2-1* mutants were a kind gift from Dr Jychian Chen (Institute of Molecular Biology, Academia Sinica, Taipei, Taiwan).

### Growth and development

Shoot biomass (fresh weight), root biomass (fresh weight), and primary root length of plantlets were measured after 15 days of cultivation on 1x MS-agar vertical plates. Chloroplastic pigments were extracted by pounding aerial parts of plantlets in 80% acetone, and absorbance of the resulting extracts was measured at 663 nm and 646 nm. Chlorophyll levels in these extracts, expressed as µg mL^−1^, were determined from the equations given by Lichtenthaler and Wellburn [53].

### Photosynthesis

Chlorophyll fluorescence and maximum PSII efficiency (F_v_/F_m_) were measured with a PAM-210 chlorophyll fluorometer system (Heinz Walz, http://www.walz.com). After dark adaptation for at least 15 min, minimum fluorescence (F_0_) was determined under weak red light. Maximum fluorescence of dark adapted leaf (F_m_) was measured under a subsequent saturating pulse of red light, and variable fluorescence (F_v_  =  F_m_−F_0_) was determined [9].

### Carbohydrate analysis

Fifteen-day-old plantlets were sampled at the end of the night (End of Night), at the middle of the day (Middle of Day), and at the end of the day (End of Day). Roots and shoots were rapidly separated and frozen in liquid nitrogen. Samples were ground to powder in liquid nitrogen and then extracted in 80% ethanol containing 4 mM HEPES-KOH, pH 7.5, at 80°C for 30 min. Samples were then centrifuged for 15 min at 11 000 *g*. This initial supernatant was collected and stored on ice. The pellets were resuspended in 80% ethanol containing 4 mM HEPES-KOH (pH 7.5), and incubated at 80°C for 30 min. After centrifugation of the extracts, for 15 min at 11 000 *g*, the supernatant was collected and stored on ice. This hot extraction of the remaining pellets was repeated further, once with 50% ethanol in 4 mM HEPES-KOH (pH 7.5) and once with 4 mM HEPES, pH 7.5. All of the resulting supernatants were then pooled and assayed for soluble sugars [54]. Quantification was carried out spectrophotometrically by enzyme-based assays using ENZYPLUS® kit (RAISIO Diagnostic, http://www.raisiodiagnostics.com).

Starch insoluble pellets were dissolved for 30 min at 60°C under agitation in dimethylsulfoxide/8M hydrochloric acid (4∶1, v/v). Samples were centrifuged for 15 min at 12 000 *g* and supernatants were used for starch determination after fixing pH at 4.5. Starch levels were determined according to Bergmeyer et al. [55] using Boehringer Mannheim enzymatic kits (ENZYPLUS®, RAISIO Diagnostic, http://www.raisiodiagnostics.com). Starch was first degraded to D-glucose by amyloglucosidase and starch concentrations were determined by quantification of the resulting D-glucose units. The D-glucose was phosphorylated to glucose-6-phosphate, and oxidized in the presence of nicotinamide adenine dinucleotide phosphate (NADP^+^) to form both gluconate-6-phosphate and NADPH. The amount of NADPH was then determined spectrophotometrically at 340 nm.

### Analysis of genomic DNA

Genomic DNA was extracted using the Wizard® Genomic DNA Purification kit (Promega, www.promega.com). Genomic DNA fragments flanking the T-DNA right (RB) and left (LB) borders were amplified by the PCR walking method described by Devic et al. [16]. Resulting PCR products were separated by electrophoresis, stained with ethidium bromide, and visualized under ultraviolet light. The amplification products were then cleaned up from agarose gel with the Nucleospin® Extract kit (Macherey-Nagel, http://www.mn-net.com), cloned into pGEM-T® (Promega, www.promega.com) and sent for sequencing at Macrogen (http://www.macrogen.com). The BLAST search program using default parameters [56] was used for sequence alignments.

Southern blot analysis was carried out as described by Hummel et al. [57]. Genomic DNA (5 µg) was independently digested with *Hin*dIII and *Nde*I restriction enzymes. T-DNA RB (591 bp) and LB (986 bp) probes were obtained by PCR of the pDB10 plasmid (Institut National de la Recherche Agronomique, Versailles, France) using respectively CGCCGATGCAGATATTCGTAAT/AGTTCATAGAGATAACCTTCACCCG and TCGTCAACCACTACATCGAGAC/CAGGATATATGCCAACGTAA as primer pairs. The resulting PCR products were sequenced and used to generate digoxigenin (DIG)-labelled DNA probes [57]. Immunodetection of hybridised probes was carried out as described in Hummel et al. [57]. Hybridization temperatures were fixed at 46°C and 50°C, respectively, for RB and LB probes.

### Quantitative RT-PCR analysis

Total RNA were extracted from plantlets using the RNAgents® Total RNA Isolation System kit (Promega, www.promega.com). One microgram of each RNA sample was reverse transcribed into cDNA with SuperScript™ II Reverse Transcriptase (BioRad, http://www.bio-rad.com). Real-time PCR amplification was carried out using a Chromo4 Real-Time PCR Detection System (Bio-Rad, http://www.bio-rad.com) and the iQ SYBR Green Supermix (Bio-Rad, http://www.bio-rad.com) as recommended by the manufacturer. Primers were designed, using Primer3 software, to be specific of *At2g21590* cDNA sequence and to amplify a sequence of approximately 150 bp. For quantification, the *ubiquitin5* (GenBank accession number: AY084978) and *β-tubulin* (GenBank accession number: AY059075) genes were used as internal controls [58]. The following pairs of primer were used:

GATTCTTCTTACTCCTTTGCCTTG/CGTGCTTGAACTTTTGATTCC for the *At2g21590* gene, CCAAGCCGAAGAAGATCAAG/TCAAAATGACTCGCCATGAA for the *ubiquitin5* gene, and ACCTTATCCCATTCCCAAGG/CAGCGGATGCAGTCAAGTAA for the *β-tubulin* gene.

### Microarray data mining and analysis

Microarray data sets were obtained from databases at The Botany Array Resource (http://bar.utoronto.ca) and come from data of the *Arabidopsis* Developmental Map [22], of the Tissue-specific root cell types [23], [24], and of the Abiotic Stress [59] and Chemical series from the AtGenExpress Consortium data. Light series from Bläsing et al. [60] and Michael et al. [61] were also used. For analysis of *APL4* and *APL3* gene expression data across the different experiments, the electronic Fluorescent Pictograph (eFP) Browser tool [62] at the Bio-Array Resource for Plant Biology website, and the Pearson correlation coefficient were used. The Pearson correlation coefficient measures the degree of association between two expression vectors. Its value is comprised between −1 and +1, inclusive, +1 meaning that the two series are identical, 0 that they are completely independent, and −1 that they are perfect opposites. Microarray analysis of *APL3* and *APL4* expression in Columbia ecotype (Col-0) under conditions of atrazine, sucrose, and atrazine plus sucrose treatment was extracted from our previous work [12]. These data are deposited in the ArrayExpress database (E-MEXP-411) according to the MIAME standards proposed by the Microarray Gene Expression Data society.

## Supporting Information

Figure S1 **Localization of T-DNA insertion in** ***eat1*** **mutant line by Southern blot analysis.** Five µg of genomic DNA from *eat1* mutant were separately digested with *Hin*dIII and *Nde*I and resulting DNA fragments were separated by agarose gel electrophoresis and then blotted onto a nylon membrane. Hybridization was carried out with specific DIG-labelled probes corresponding to T-DNA left and right borders. Southern blot analysis was carried out as previously described [57].
(PPT)

Figure S2 **Enhanced biomass phenotype of BH755830 Arabidopsis mutant line.** Root and shoot fresh weights are given. Seeds of the BH755830 Arabidopsis mutant line were germinated on 1x MS-agar medium in the absence of atrazine, and plantlet development was carried out for 15 days. Values are the mean (± S.E.M.) of measurements on at least sixteen 15-day-old plantlets. Asterisks represent statistically significant differences (Mann-Whitney test, P<0.05) between WT (Col-0) and BH755830.
(PPT)
