# Supplementary figures and images for: Carbon Dynamics, Development and Stress Responses in Arabidopsis: Involvement of the APL4 Subunit of ADP-Glucose Pyrophosphorylase (Starch Synthesis)
Source: PLoS One. 2011 Nov 3;6(11):e26855. doi: 10.1371/journal.pone.0026855 (PMC3207819; doi:10.1371/journal.pone.0026855)

## Slide 1
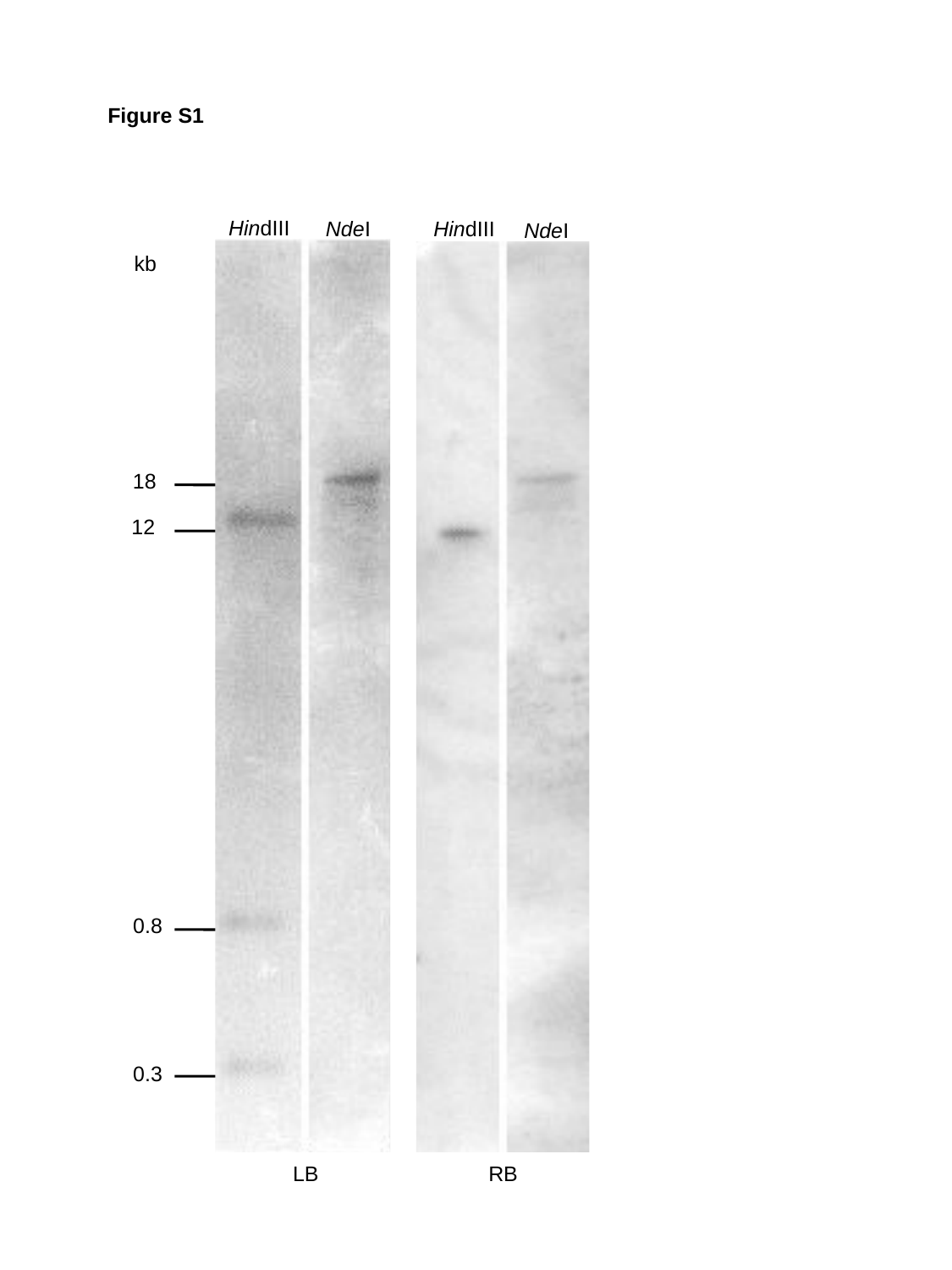

Figure S1
HindIII
NdeI
HindIII
NdeI
kb
18
12
0.8
0.3
LB
RB

Supplement: Figure S1 — Localization of T-DNA insertion in eat1 mutant line by Southern blot analysis. Five µg of genomic DNA from eat1 mutant were separately digested with HindIII and NdeI and resulting DNA fragments were separated by agarose gel electrophoresis and then blotted onto a nylon membrane. Hybridization was carried out with specific DIG-labelled probes corresponding to T-DNA left and right borders. Southern blot analysis was carried out as previously described [57]. (PPT) [file pone.0026855.s001.ppt]

## Slide 1
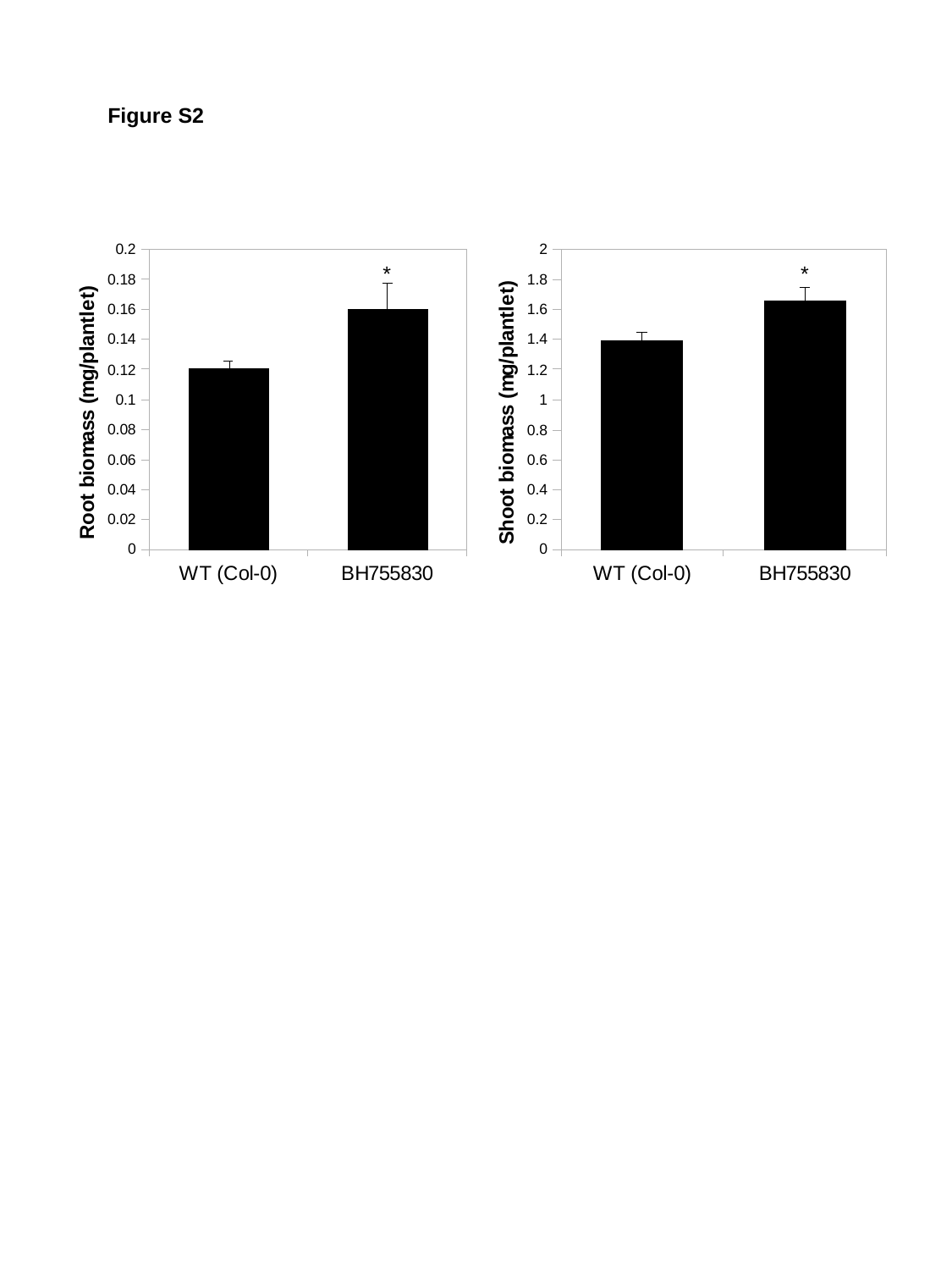

Figure S2
*
*

Supplement: Figure S2 — Enhanced biomass phenotype of BH755830 Arabidopsis mutant line. Root and shoot fresh weights are given. Seeds of the BH755830 Arabidopsis mutant line were germinated on 1x MS-agar medium in the absence of atrazine, and plantlet development was carried out for 15 days. Values are the mean (± S.E.M.) of measurements on at least sixteen 15-day-old plantlets. Asterisks represent statistically significant differences (Mann-Whitney test, P<0.05) between WT (Col-0) and BH755830. (PPT) [file pone.0026855.s002.ppt]
